# Detecting and characterizing new endofungal bacteria in new hosts: *Pandoraea sputorum* and *Mycetohabitans endofungorum* in *Rhizopus arrhizus*

**DOI:** 10.3389/fmicb.2024.1346252

**Published:** 2024-02-29

**Authors:** Xiao-Ling Liu, Heng Zhao, Yi-Xin Wang, Xin-Ye Liu, Yang Jiang, Meng-Fei Tao, Xiao-Yong Liu

**Affiliations:** ^1^College of Life Sciences, Shandong Normal University, Jinan, China; ^2^State Key Laboratory of Mycology, Institute of Microbiology, Chinese Academy of Sciences, Beijing, China; ^3^State Key Laboratory of Efficient Production of Forest Resources, School of Ecology and Nature Conservation, Beijing Forestry University, Beijing, China

**Keywords:** *Rhizopus oryzae*, novel endosymbiont, endohyphal bacterium, *Pandoraea sputorum*, comparative genomics

## Abstract

The fungus *Rhizopus arrhizus* (=*R. oryzae*) is commonly saprotrophic, exhibiting a nature of decomposing organic matter. Additionally, it serves as a crucial starter in food fermentation and can act as a pathogen causing mucormycosis in humans and animals. In this study, two distinct endofungal bacteria (EFBs), associated with individual strains of *R. arrhizus*, were identified using live/dead staining, fluorescence *in situ* hybridization, transmission electron microscopy, and 16S rDNA sequencing. The roles of these bacteria were elucidated through antibiotic treatment, pure cultivation, and comparative genomics. The bacterial endosymbionts, *Pandoraea sputorum* EFB03792 and *Mycetohabitans endofungorum* EFB03829, were purified from the host fungal strains *R. arrhizus* XY03792 and XY03829, respectively. Notably, this study marks the first report of *Pandoraea* as an EFB genus. Compared to its free-living counterparts, *P. sputorum* EFB03792 exhibited 28 specific virulence factor-related genes, six specific CE10 family genes, and 74 genes associated with type III secretion system (T3SS), emphasizing its pivotal role in invasion and colonization. Furthermore, this study introduces *R. arrhizus* as a new host for EFB *M. endofungorum*, with EFB contributing to host sporulation. Despite a visibly reduced genome, *M. endofungorum* EFB03829 displayed a substantial number of virulence factor-related genes, CE10 family genes, T3SS genes, mobile elements, and significant gene rearrangement. While EFBs have been previously identified in *R. arrhizus*, their toxin-producing potential in food fermentation has not been explored until this study. The discovery of these two new EFBs highlights their potential for toxin production within *R. arrhizus*, laying the groundwork for identifying suitable *R. arrhizus* strains for fermentation processes.

## Introduction

Bacteria residing within the vegetative or reproductive structures of fungi are referred to as endofungal or endohyphal bacteria (EFBs or EHBs), representing one of the most intricate relationships between bacteria and fungi (Deveau et al., 2018; Pawlowska et al., 2018). The presence of EFBs was initially reported by Mosse (1970) through electron microscopy in the cytoplasm of *Endogone* spores. In the following decades, researchers discovered EFBs in various species of arbuscular mycorrhizal fungi (AMF), distinguishing two shapes (rod-shaped and irregularly coccoid; MacDonald and Chandler, 1981; Sward, 1981; Scannerini and Bonfante-Fasolo, 1991; Schüßler et al., 1994). EFBs were later identified using bacteria-specific dyes, fluorescence *in situ* hybridization (FISH) with bacteria-specific probes, and pyrosequencing (Sun et al., 2019). They were categorized into facultative and obligate based on their *in vitro* cultivability (Mondo et al., 2012; Bonfante and Desirò, 2017; Uehling et al., 2017, 2023). The symbiotic relationship between EFBs and host fungi involves mutualistic benefits and occasional antagonism (Lastovetsky et al., 2020; Venkatesh et al., 2022), impacting asexual and sexual reproduction of the host fungi (Partida-Martinez et al., 2007c; Mondo et al., 2017). This symbiosis extends to form a tripartite interaction with plants or animals, contributing to plant or animal health and performance (Desirò et al., 2015; Guo and Narisawa, 2018; Büttner et al., 2023; Cappelli et al., 2023). The EFB-fungi interaction has gained attention due to its relevance to agriculture and industry.

Many EFBs are associated with the fungal phylum Mucoromycota, and belong to Betaproteobacteria (Burkholderia-related endobacteria, BREs) and *Mollicutes* (*Mycoplasma*-related endobacteria, MREs) (Pawlowska et al., 2018; Okrasińska et al., 2021; Richter et al., 2022; Uehling et al., 2023). The BRE *Mycetohabitans rhizoxinica*, highly dependent on its host *R. microsporus*, was protected by transcription activator-like (TAL) effectors, while produced toxins rhizoxin and rhizonin with implications in causing rice seedling blight disease and hepatotoxicity (Partida-Martinez and Hertweck, 2005; Partida-Martinez et al., 2007a; Richter et al., 2023). More EFBs of Mucoromycota demonstrate significant potential in biosynthesizing secondary metabolites and activating fungal genes related to toxin synthesis and pathogenicity (Muszewska et al., 2021; Cheng et al., 2022; Niehs et al., 2022; Ghasemi et al., 2023). However, the chemical signals involved in these interactions remain poorly understood, posing potential risks to third parties beyond bacteria and host fungi (Zhou et al., 2022).

The genus *Rhizopus*, characterized by abundant rhizoids on hyphae and stolons, encompasses 12 species widely distributed in soil and air, playing key roles in industrial, agricultural, and medical applications (Zheng et al., 2007; Liu et al., 2019; Zhao et al., 2023). Notably, *R. arrhizus* and *R. microsporus* are crucial in food fermentation and mucormycosis (Cheng et al., 2017; Yao et al., 2018; Rudramurthy et al., 2023), presenting concerns about the role of EFBs in mucormycosis infection, especially in the context of COVID-19 complications (Dogra et al., 2022). Recent studies have screened EFB-free strains of *R. arrhizus* to ensure food safety during fermentation (Hamza and Gunyar, 2022). The impact of EFB *Ralstonia pickettii* in *R. microsporus* on phagocyte evasion and opportunistic virulence has been reported (Itabangi et al., 2022). The presence of EFB *Mycetohabitans rhizoxinica* in a cancer patient further emphasizes the role of endosymbionts in the virulence of their host fungus *R. microsporus* (Yang et al., 2022). While mucormycosis is usually caused by co-infection of *R. arrhizus* and *R. microsporus*, the contribution of EFB to the pathogenesis of *R. arrhizus* remains to be confirmed.

To date, five EFB species have been detected in *Rhizopus arrhizus*, including two unnamed BREs and three named Gammaproteobacteria (*Serratia marcescens*, *Pseudomonas fluorescens*, and *Klebsiella pneumoniae*; Ibrahim et al., 2008; Itabangi et al., 2019; Birol and Gunyar, 2021). In this study, we confirmed the presence of two Burkholderiaceae EFBs in different parts of *R. arrhizus* through 16S rDNA sequencing and microscopic observation. To assess their potential impact on food safety and toxin production, we conducted a comparative analysis of their genetic background through whole-genome resequencing.

## Materials and methods

### Strains

This study utilized two strains, XY03792 and XY03829, of *Rhizopus arrhizus*. The XY03792, sourced from soy sauce in Malaysia, is a fermentative strain. The XY03829 is a wild strain obtained from animal dung in Pakistan. Both strains demonstrated the capability to ferment, resulting in the production of various compounds such as maltose, glucose, ethanol, lactic acid, fumaric acid, malic acid, glycerol, among others (Yao et al., 2018; Liu et al., 2022). These strains were preserved at Shandong Normal University under −20°C with 15% glycerine.

### Manipulation and cultivation

To prevent bacterial contamination, sporangiospores underwent a meticulous two-step surface sterilization process following the method outlined by Becard and Fortin (1988). In the initial step, sporangiospores were immersed in a 0.05% Tween 20 solution for two minutes, followed by a 10 min soak in a 2% chloramine T solution. Subsequently, they were thoroughly rinsed three times with sterile distilled water. This soak and wash procedure was repeated once more, after which the sporangiospores were preserved in a sterile solution containing 200 mg/L streptomycin and 100 mg/L gentamicin at 4°C. Moving to the second step, the stored sporangiospores underwent another round of soaking in a 2% chloramine T solution and were washed with sterile distilled water immediately before inoculation. The surface-sterilized sporangiospores, treated through this two-step process, were then cultured on potato dextrose agar (PDA: 200 g/L potato, 20 g/L glucose, 20 g/L agar, and 1,000 mL distilled water) at 30°C.

### DNA extraction, PCR, and sanger sequencing

To avoid bacterial contamination during incubation, the mycelia cultivated on PDA for 5 days underwent surface sterilization using 30% hydrogen peroxide, following the procedure outlined by Izumi et al. (2006). Metagenomic DNAs of *Rhizopus arrhizus* and its EFBs were extracted using the GOMag Rapid Plant DNA Kit (GO-GPLF-400, GeneOn BioTech, China). According to the manufacturer’s instructions, approximately 30 mg of thalli were successively lysed, adsorbed, washed, and eluted for metagenome extraction. An empty centrifuge tube served as a negative control. The 16S rDNA was amplified using primers 27F (5′-AGA GTT TGA TCC TGG CTC AG-3′) and 1541R (5′-AAG GAG GTG ATC CAG CC-3′). The PCR mixture (25.0 μL) included 1.0 μL of template DNA (10.0 ng/μL), 1.0 μL of the two primers each (10.0 μM), 12.5 μL of 2 × Taq PCR Master Mix (Biomed Diagnostics Pte Ltd., Singapore), and 9.5 μL of sterile deionized water (Caporaso et al., 2012). PCR amplification involved an initial step at 94°C for 5 min, followed by 35 cycles of 94°C for 30 s, 55°C for 30 s, and 72°C for 1 min, with a final extension at 72°C for 10 min. Sanger sequencing was carried out using the same primers (27F and 1541R) as used in PCR. Phylogenetic reconstruction employed the maximum likelihood (ML) and Bayesian inference (BI) methods through RAxML version 8.1.5 and MrBayes 3.2.7a, respectively (Ronquist et al., 2012; Stamatakis, 2014). Bootstrap supports (BS) for branches were obtained through 1,000 replicates (Estrada-De Los Santos et al., 2018). The resulting tree was edited online using the interactive Tree of Life platform (iTOL, https://itol.embl.de/itol.cgi; Letunic and Bork, 2019).

### Visualizing EFB by microscopic observation

#### Live/dead staining

The Live/dead BacLight Bacterial Viability Kit (catalogue number L7012, Invitrogen, United States) was employed for the initial detection of EFBs following the method outlined by Arendt et al. (2016) and Takashima et al. (2018). Fresh hyphae and sporangiospores, obtained by scraping from the fungal colony on PDA, were deposited onto a glass slide along with 15.00 μL of a 1:1:200 mixed stain solution (SYTO9: propidium iodide: sterile 0.85% NaCl). Subsequently, cover slips were mounted onto the slide, and the preparation was incubated at room temperature in darkness for a few minutes. The stained hyphae and sporangiospores were then examined using an inverted fluorescence microscope (Axio observer Z1, Zeiss, Germany).

#### Fluorescence *in situ* hybridization

A probe (5′-CTT CCG GTA CCG TCA TCC CCC CGA GG-3′) labeled with Invitrogen Cyanine3 (Cy3) dye was designed for fluorescence *in situ* hybridization (FISH), targeting the 16S rDNA sequences specific to *Pandoraea sputorum*. FISH procedures were conducted following the method outlined by Hoffman and Arnold (2010). The general steps were as follows: Mycelia cultivated on PDA for 3 days were fixed at 4°C for 3 h using a 3:1 mixed fix solution of formalin (10%) and phosphate-buffered saline (PBS). The fixed mycelia were washed twice with PBS buffer and subsequently dehydrated with 50, 70, and 95% ethanol. The mycelia were then incubated with 8 μL of a 40% formamide hybridization stringency solution (800 μL formamide, 800 μL diethyl pyrocarbonate water, 500 μL 5 M EDTA) and 2 μL of the probe (10 μM) at 46°C for 1.5 h. Each sample underwent rinsing with 100 μL of wash buffer (460 μL 5 M NaCl, 1,000 μL 1 M Tris, 50 μL 10% SDS, made up to 50 mL with diethyl pyrocarbonate water) at 46°C. Fungal DNA was stained with 10 μL of 4,6-diamidine-2-phenylindole dihydrochloride (DAPI, Sigma) for 10 min and subsequently removed by washing with distilled water. Fluorescence images were captured using an inverted fluorescence microscope (Axio observer Z1, Zeiss, Germany). For the Cy3-labelled probe, the excitation and emission wavelengths were 550 nm and 580 nm, respectively (Naumann et al., 2010). For DAPI staining, the excitation and emission wavelengths were 358 nm and 461 nm, respectively (Guo et al., 2017).

#### Transmission electron microscopy

To precisely determine their specific location within the mycelium, EFB were visualized using transmission electron microscopy (TEM). A small mycelial pellet from a 3 days-old culture of *Rhizopus arrhizus* XY03829 was fixed with 0.1% glutaraldehyde/4% paraformaldehyde in 1× Phosphate Buffered Saline (PBS, pH 7.0) for 1 h at 25°C and subsequently overnight at 4°C. The pellets were then embedded in a drop of water agar and subjected to five washes with 1 × PBS. Further fixation was performed with a 1% (w/v) osmium tetraoxide (OsO4) solution for one hour. After three rinses with 1 × PBS, the samples underwent sequential dehydration in an ethanol series and were then immersed three times in 100% acetone. For infiltration, the samples were treated with a 3:1 acetone-resin mixture for 0.5 h, 1:1 for 1 h, and 1:3 for 1.5 h. Subsequently, the fungal samples were embedded in fresh Spurr resin and polymerized for 12 h at 70°C. Ultrathin sections were cut using an ultramicrotome (EM FC7, LEICA) and stained with uranyl acetate and lead citrate. The grids were examined using a JEM-1400Plus transmission electron microscope with an EM-14830RUBY2 charge-coupled device (CCD) camera (JEOL, Tokyo, Japan) at an acceleration voltage of 100 kV.

### Isolation and identification of EFB

To isolate endosymbiotic bacteria, the host fungi were cultivated on PDA at 28°C for 3 days. A pellet of thalli, sterilized glass beads, and 1 mL lysogeny broth (LB: 10 g/L tryptone, 5 g/L yeast extract, 10 g/L NaCl) were added to a 2 mL centrifuge tube. The mixture was homogenized using a high-throughput tissue grinder (SCIENTZ-48) at 45 Hz for 30 s. The homogenized tissue fluid was then filtered through a 5 μM membrane and spread on lysogeny agar (LA: 10 g/L tryptone, 5 g/L yeast extract, 10 g/L NaCl, agar 30 g/L). Finally, it was incubated at 30°C for 7 days, and a single colony was transferred to another LA plate.

### EFB genome sequencing and comparative genomic analysis

A single colony of EFBs grown on LA plates was inoculated into 15 mL of LB medium and shaken at 180 rpm at 37°C for 18 h. Bacterial cells were collected by centrifugation, and genomic DNAs were extracted using the Wizard Genomic DNA Purification Kit (A1120) following the manufacturer’s instructions. DNA integrity was verified on an agarose gel. Whole-genome resequencing was performed on the BENAGEN platform using Nanopore and Illumina NovaSeq PE150 platforms.

Raw data were assessed using FastQC 0.11.8 (Andrews, 2010) and Trimmomatic 0.39 (Bolger et al., 2014) for the filtration of low-quality reads, resulting in clean reads. The clean reads were assembled using MaSuRCA 3.4.3b (Zimin et al., 2013) and SPAdes 3.14 (Bankevich et al., 2012). Gene-coding models were predicted with Prokka (Seemann, 2014). For gene functional annotation, the predicted models were compared to various databases, including UniProt,^1^ NR,^2^ Pfam,^3^ KEGG,^4^ GO,^5^ CAZy,^6^ COG,^7^ CARD,^8^ and VFDB.^9^ Prophages, insertional sequences, and gene islands were predicted using PHASTER,^10^ ISFinder,^11^ and IslandViewer^12^, respectively.

Transposase and integrase sequences were sourced from the NCBI protein database, followed by clustering and classification using transposon and integron annotation databases, respectively. Initially, the Diamond blast+ software (version 0.9.31; Buchfink et al., 2015) was employed to compare genome and protein sequences against the Uniprot database. The outcomes of this comparison were integrated with the pre-constructed transposon and integron annotation databases to facilitate the prediction of transposons and integrons. Genomic collinearity analysis was conducted using the MAUVE (Darling et al., 2004). Subsequently, based on the results obtained from the Comprehensive Antibiotic Resistance Database (CARD) and the Virulence Factor Database (VFDB), a Venn diagram illustrating differences in gene numbers was generated using the Venny website.^13^

Two free-living *Pandoraea sputorum* strains, NCTC13161 (BioProject ID: PRJEB6403) and DSM21091 (BioProject ID: PRJNA262705), were downloaded from the NCBI database as references for *P. sputorum* EFB03792. The genome of EFB *Mycetohabitans endofungorum* HKI456 (BioProject ID: PRJNA370785) was used as the reference for *M. endofungorum* EFB03829.

### Curing fungal strains and co-culturing with free-living EFB

The strains, preserved at −20°C with 15% glycerine, were inoculated on PDA plates supplemented with 100 μg/mL ampicillin, 50 μg/mL kanamycin, 10 μg/mL tetracycline, and 40 μg/mL ciprofloxacin. The plates were then incubated at 28°C for 36 h. Subcultures were performed under the same conditions for 30 generations. In each generation, the strain’s morphology was documented through photography and verified using live/dead staining. The cured fungal strain and free-living EFB isolated from the corresponding wild-type strain were simultaneously inoculated at the same position on lysogeny agar (LA) plates to observe whether the cured fungi resumed sporulation.

## Results

### Molecular detection and identification of EFBs

**Figure 1:**
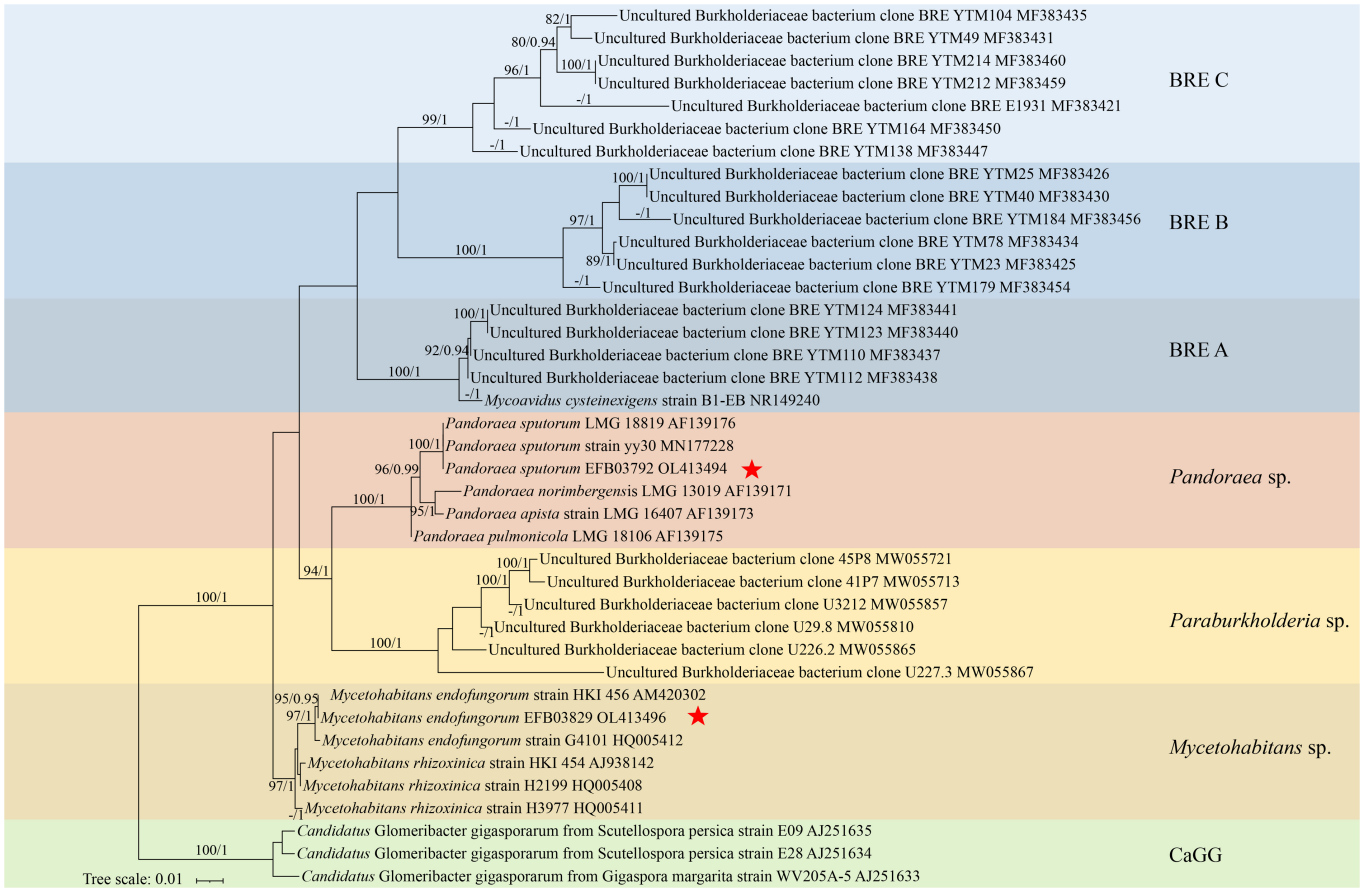
A maximum likelihood consensus phylogenetic tree of *Burkholderia*-related endobacteria (BRE) showing the placement of *Pandoraea sputorum* EFB03792 and *Mycetohabitans endofungorum* EFB03829. The *Candidatus* Glomeribacter gigasporarum was used as outgroup. All nodes with maximum likelihood bootstrap values (MLBV) and Bayesian inference posterior probabilities (BIPP) >80% and >0.90 are successively labelled and separated by a slash “/.” Sequences obtained herein are marked with a red star “*.” GenBank accession numbers are shown after the species name. Background colours indicate groups [blue, BRE **(A–C)**; red, *Mycetohabitans* spp.; yellow, *Paraburkholderia* spp.; brown, *Pandoraea* spp.; green, *Candidatus* Glomeribacter gigasporarum]. The lower left bar represents 0.01 expected substitutions per site.

The 16S rDNA was successfully amplified from the metagenome of the two strains of the fungus *Rhizopus arrhizus*, resulting in a target fragment with a length of approximately 1.5 kb. These sequences were deposited in GenBank under the accession numbers OL413494 and OL413496. Identical 16S rDNA sequences were also annotated from the metagenome of the corresponding fungal strains. The maximum likelihood phylogenetic tree of EFBs based on 16S rDNA sequences is presented in Figure 1. In this phylogram, the two EFBs individually residing in the *R. arrhizus* strains XY03792 and XY03829 were grouped into the clades *Pandoraea sputorum* and *Mycetohabitans endofungorum*, respectively. Specifically, *P. sputorum* exhibited a close relationship with *P. apista* and *P. norimbergensis*, while *M. endofungorum* was closely related to *M. rhizoxinica*.

### *In situ* detection of EFBs

**Figure 2:**
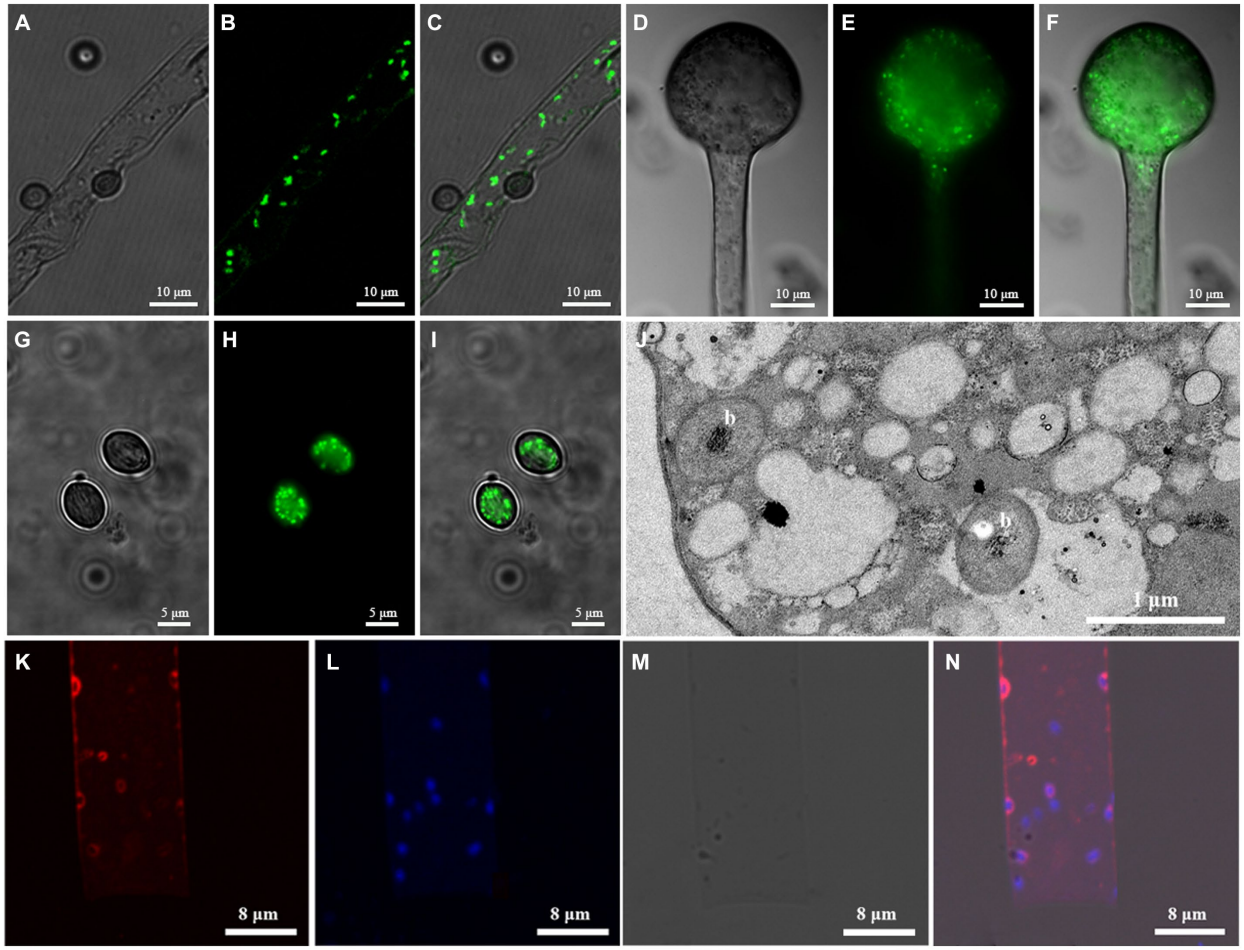
Microscopic images showing EFB living in *Rhizopus arrhizus* XY03792 and XY03829. **(A–I)** Live/dead staining images showing EFB living in XY03829. **(A-C)** Hyphae; **(D-F)** Columellae; **(G-I)** Sporangiospores; A/D/G, SYTO-9; B/E/H, DIC (Differential interference contrast); (C/F/I), Mixed image; **(J)** Transmission electron microscopy (TEM) images of EFB (marked with letter b) living in the mycelia of XY03829; **(K–N)** Fluorescence *in situ* hybridization images showing EFB living in XY03792. **(K)** Cy3; **(L)** DAPI; **(M)** DIC; **(N)** Mixed image.

Following live/dead staining, EFBs with green fluorescence were observed within the hyphae, columellae, and sporangiospores of both fungal strains (Figures 2A–I). Fluorescence *in situ* hybridization (FISH) revealed red fluorescence in the hyphae of *Rhizopus arrhizus* XY03792 (Figures 2K–N), while green fluorescence was observed in the hyphae of *R. arrhizus* XY03829 (data not shown), confirming the presence of specific EFBs. Transmission electron microscopy (TEM) images showed clear transverse sections of bacterial rods (Figure 2J), indicating the localization of EFBs within the cytoplasm of fungal mycelia rather than in vacuoles. The bacteria within the cytosol were distinguishable from fungal organelles due to their visible cell walls, nucleoids, reserve materials, and morphology. The cross-section size of these cells ranged from 0.6 to 0.8 μm in diameter.

### General genome features of EFBs

**Figure 3:**
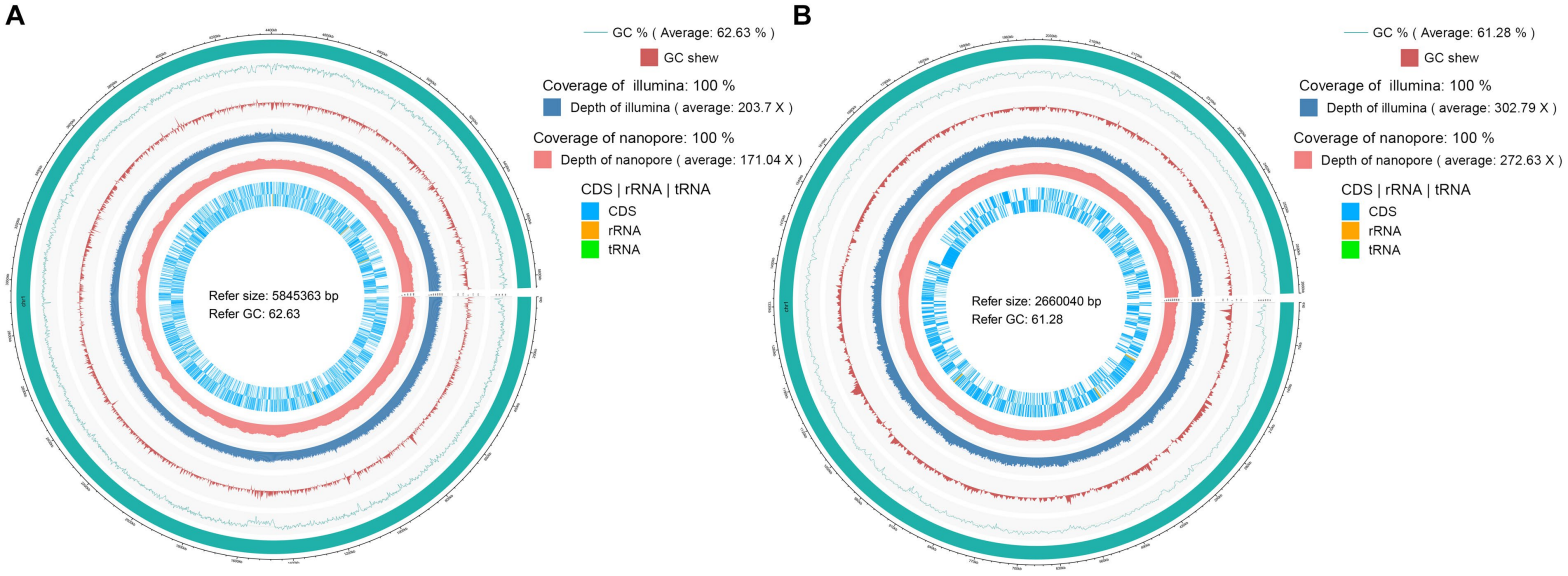
Circular maps of the complete genomes of two EFB associated with *Rhizopus arrhizus*. **(A)** *Pandoraea sputorum* EFB03792; **(B)** *Mycetohabitans endofungorum* EFB03829.

The genome of *Pandorea sputorum* EFB03792 comprised approximately 1.00 Gb of clean data from the Nanopore sequencing platform, encompassing 22,138 reads with an average sequence read length of 45,173 bp, achieving full coverage (100%) and an average sequencing depth of around 171×. The Illumina-filtered clean data amounted to approximately 1.19 Gb, encompassing 7,962,964 reads with an average sequence read length of 150 bp, achieving full coverage (100%) and an average sequencing depth of approximately 203×. The assembled genome contained one circular chromosome spanning 5,845,363 bp with a GC content of 62.63%. A total of 5,215 gene models were encoded, including 5,099 coding sequences (CDS), 75 transfer RNA (tRNA), 12 ribosomal RNA (rRNA), and two transfer-messenger RNA (tmRNA) (Figure 3A and Table 1).

**Table 1:** Genomic features of *Pandoraea sputorum* and *Mycetohabitans endofungorum* sequenced and *de novo* assembled in this study.

Species		*P. sputorum* EFB03792	*M. endofungorum* EFB03829
Genome size (bp)		5,845,363	2,660,040
Chromosome		1	1
Plasmid		0	2
GC (%)		62.63	47.46
Gene models		5,215	3,359
	Uniprot	3,365	1,659
	Pfam	4,612	2,687
	NR	5,076	3,159
	COG	2,239	1,267
	KEGG	2,721	1,761
	GO	3,275	1,852
	CAZymes	84	65
	CARD	39	35
	VFDB	455	299
RNA			
	tRNA	75	48
	rRNA	12	9
	tmRNA	2	1
Numbers prophages		2	1
Numbers genomics islands		9	25
Repetitive elements (% in genomes)		0.57	0.66

The genome of *Mycetohabitans endofungorum* EFB03829 consisted of approximately 1.00 Gb of clean data from the Nanopore sequencing platform, encompassing 30,159 reads with an average sequence read length of 33,158 bp, achieving full coverage (100%) and an average sequencing depth of around 273×. The Illumina-filtered clean data amounted to 1.14 Gb, encompassing 7,598,064 reads with an average sequence read length of 150 bp, achieving full coverage (100%) and an average sequencing depth of approximately 303×. The assembled genome comprised one circular chromosome spanning 2,660,040 bp with a GC content of 61.28%. A total of 3,359 gene models were encoded, including 3,268 CDS, 48 tRNA, nine rRNA, and one tmRNA (Figure 3B and Table 1). Additionally, two plasmids were assembled, with Plasmid 1 measuring 800,149 bp long and exhibiting a GC content of 59.61%, and Plasmid 2 measuring 181,953 bp long with a GC content of 57.63%.

### Functional annotations of genomes

Among the 5,215 gene models of the strain EFB03792 of *Pandorea sputorum*, 3,365, 4,612, 5,076, 2,239, 2,721, 3,275, 84, 39, and 455 genes were annotated with the UniProt, Pfam, NR, COG, KEGG, GO, CAZy, CARD, and VFDB databases, respectively (Table 1 and Supplementary File S1). In the case of *Mycetohabitans endofungorum* EFB03829, out of 3,359 gene models, 1,659, 2,687, 3,159, 1,267, 1,761, 1,852, 65, 35, and 299 genes were annotated with the UniProt, Pfam, NR, COG, KEGG, GO, CAZy, CARD, and VFDB databases, respectively (Table 1 and Supplementary File S2).

The COG annotation results (Supplementary Figure S1) indicated that *M. endofungorum* EFB03829 had fewer genes in all groups compared to *P. sputorum* EFB03792, except for mobile genes, which were more in EFB03792 than in EFB03829 (35 vs. 12, Supplementary Figure S1).

In terms of CAZy annotation, *P. sputorum* EFB03792 possessed 12 auxiliary activity genes (AAs), five carbohydrate-binding module genes (CBMs), 13 carbohydrate esterase genes (CEs), 19 glycoside hydrolase genes (GHs), 34 glycosyl transferase genes (GTs), and one polysaccharide lyase gene (PL; Supplementary File S3). *M. endofungorum* EFB03829 completely lost CBM and PL genes but had four AAs, five CEs, 18 GHs, and 38 GTs (Supplementary File S3).

CARD annotation revealed that *P. sputorum* EFB03792 possessed a unique drug resistance gene, specifically the FAD-containing monooxygenase EthA, which confers resistance to ethionamide through antibiotic target alteration. There were no differences in drug resistance genes between *M. endofungorum* EFB03829 and HKI45.

**Figure 4:**
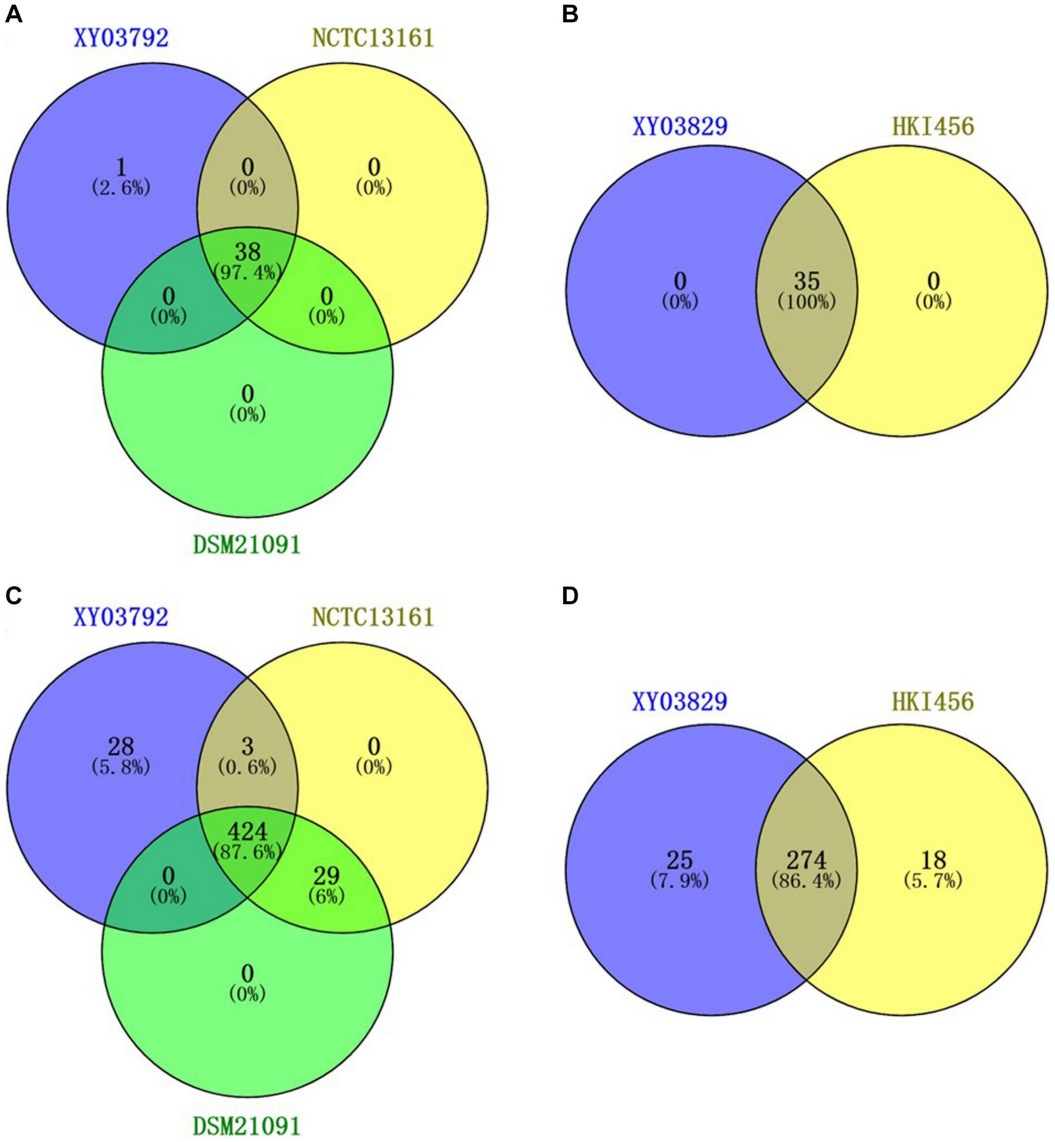
Venn diagram of different genes based on CARD **(A,B)** and VFDB **(C,D)** annotation. **(A,C)** *Pandoraea sputorum* strains EFB03792, NCTC13161, and DSM21091; **(B,D)** *Mycetohabitans endofungorum* strains EFB03892 and HKI456.

VFDB annotation results showed that *P. sputorum* EFB03792 and *M. endofungorum* EFB03829 had 656 and 309 virulence factor-related genes, respectively (Supplementary Files S4, S5). Among these genes, 28 were specific in *P. sputorum* EFB03792 and 25 were specific in *M. endofungorum* EFB03829 (Figures 4C,D and Supplementary Table S1).

Additionally, two prophages were predicted in *P. sputorum* EFB03792, and one was predicted in *M. endofungorum* EFB03829 (Table 1). Nine and 25 genomic islands were annotated in *P. sputorum* EFB03792 and *M. endofungorum* EFB03829, respectively (Table 1).

### Gene structure of EFBs

**Figure 5:**
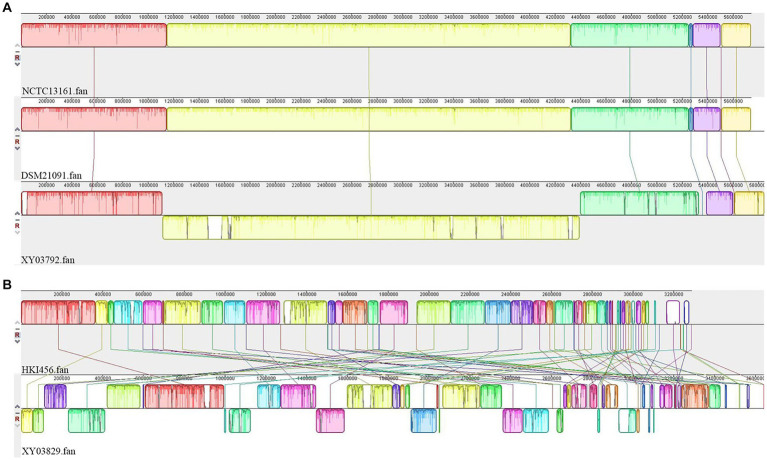
Collinearity analyses of EFB genomes. **(A)** *Pandoraea sputorum*; **(B)** *Mycetohabitans endofungorum*.

For *Pandorea sputorum*, compared with the free-living strains NCTC13161 and DSM21091, the endosymbiotic strain EFB03792 exhibited an inversion in the structure for more than half of its genes (Figure 5A) and contained a higher number of mobile elements (111 vs. 60; Table 1).

Concerning *Mycetohabitans endofungorum*, in comparison with the obligate endosymbiotic strain HKI456, the facultative endosymbiotic strain EFB03829 displayed a significant number of genes involved in inversion and/or translocation (Figure 5B) and contained a much larger number of mobile elements (1,517 vs. 392; Table 2).

**Table 2:** Numbers of mobile genetic elements in *Pandoraea sputorum* and *Mycetohabitans endofungorum*.

	Plasmid	Prophage	Insertion sequence	Genomics island	Transposon	Total
*P. sputorum* EFB03792	0	1	92	9	9	111
*P. sputorum* NCTC13161	0	2	49	8	1	60
*P. sputorum* DSM21091	0	2	49	8	1	60
*M. endofungorum* EFB03829	2	10	1,389	25	94	1,517
*M. endofungorum* HKI456	0	6	361	14	11	392

### CAZy analyses in EFBs

**Figure 6:**
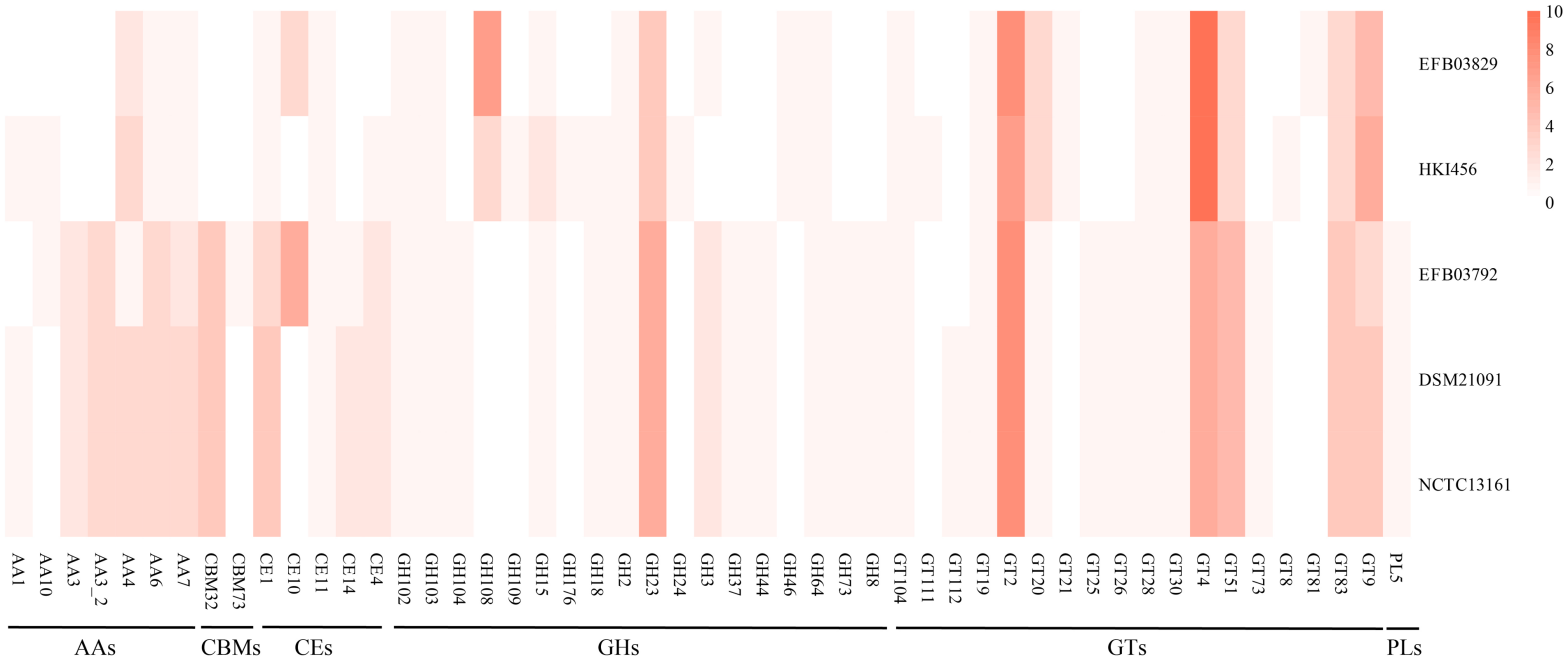
The genes number of CAZymes form *Mycetohabitans endofungorum* (EFB03829 and HKI456) and *Pandoraea sputorum* (EFB03792, DSM21091, and NCTC13161).

In this study, CAZy annotation was performed on five strains, namely *Mycetohabitans endofungorum* EFB03829, HKI456, *Pandoraea sputorum* EFB03792, DSM21091, and NCTC13161. The newly assembled genomes of *M. endofungorum* EFB03829 and *P. sputorum* EFB03792 were predicted with three and six CE10 family genes, respectively, while the other three genomes lacked (Figure 6). The CE10 family genes encoded some enzymes that catalyzed the hydrolysis of carboxylic ester bonds, such as acetyl-hydrolase, monoterpene epsilon-lactone hydrolase, acetyl esterase/lipase, and carboxylesterase.

Fewer CAZy genes were identified in *M. endofungorum* (65–67) than in *P. sputorum* (84). *Mycetohabitans endofungorum* exhibited a complete loss of CBM (CBM32 and CBM73) and PL5 family genes compared to *P. sputorum* (Figure 6 and Supplementary File S3). The CBM32, CBM73, and PL5 families encoded beta-galactosidase, chitin binding, and alginate lyase, respectively. All strains possessed a rich abundance of CEs, GHs, and GTs (e.g., CE1, GH23, GT4, GT83, and GT9 family) genes (Figure 6). GH108 family genes were enriched in *M. endofungorum* (seven in EFB03829 and three in HKI456), but none in *P. sputorum*. These GH108 family genes were presumed to have a putative peptidoglycan binding domain and a predicted peptidoglycan domain.

### Type III secretion system predicted in EFBs

In this study, 24 and 74 genes related to the type III secretion system (T3SS) were predicted from *Mycetohabitans endofungorum* EFB03829 and *Pandorea sputorum* EFB03792, respectively (Supplementary File S6), and *M. endofungorum* EFB03829 and *P. sputorum* EFB03792 have completely T3SS. The T3SS of *M. endofungorum* EFB03829 have five ATPase complexes, two basal bodies, four cytoplasmic rings, three export apparatuses, eight regulators, and two invasion protein genes. The T3SS of *P. sputorum* EFB03792 exhibited five ATPase complexes, eight basal bodies, six cytoplasmic rings, six export apparatuses, 45 regulators, and five invasion protein genes.

### Morphological changes of cured fungal strains

**Figure 7:**
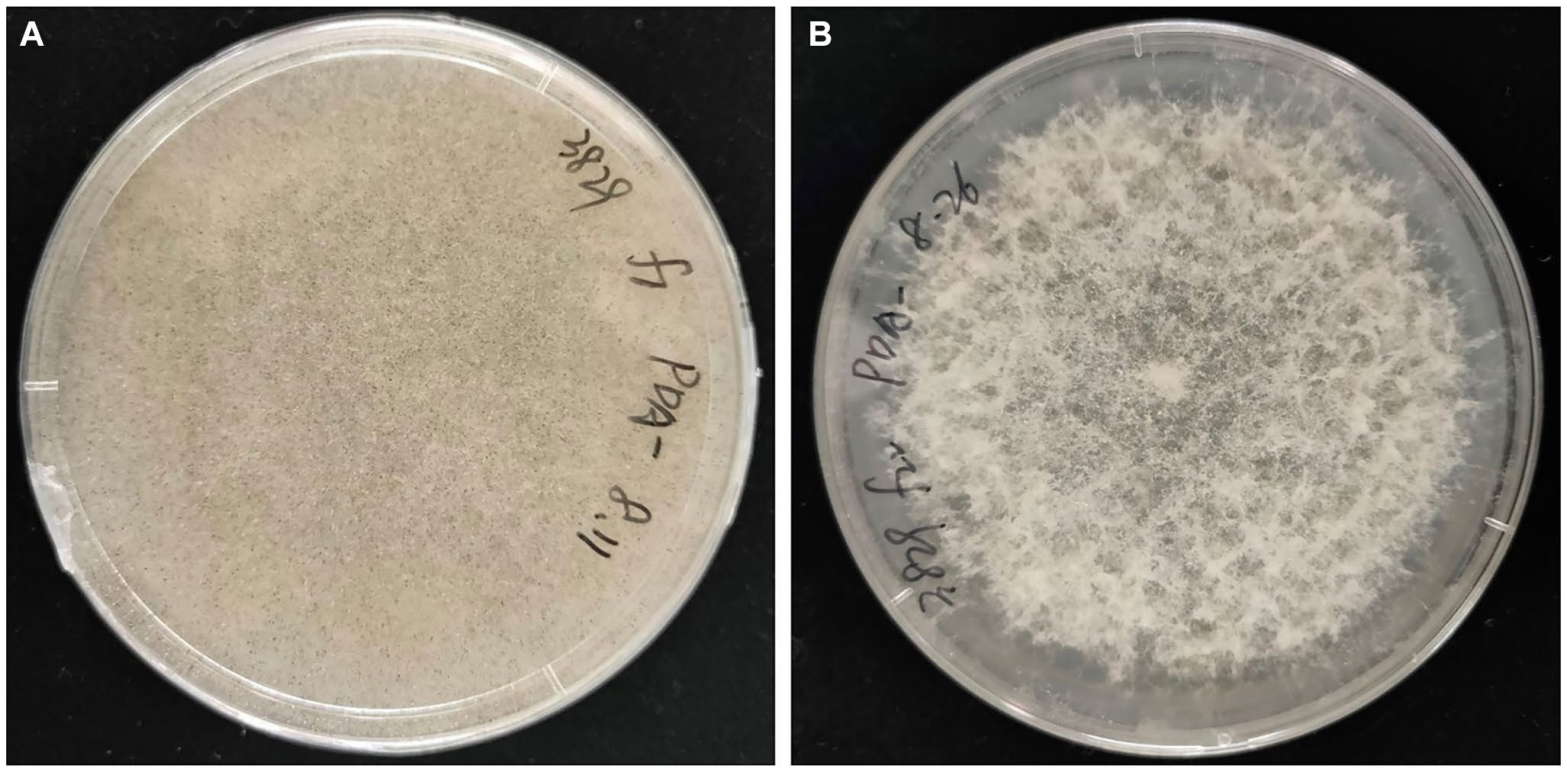
Mycelial morphologies of *Rhizopus arrhizus* XY03829. **(A)** Without antibiotic treatments; **(B)** Undergoing antibiotic treatments.

*Rhizopus arrhizus* underwent continuous sub-culturing on a PDA plate containing antibiotics. Throughout the subculturing process, the sporangiophores of the strain XY03829 exhibited increased bending, and the production of sporangiospores gradually decreased. Starting from the 22nd generation, no sporangiospores were formed, and the strain could not recover to produce any sporangiospores (Figure 7). In contrast, the mycelial morphology of *R. arrhizus* XY03792 remained unchanged during the subculturing process.

## Discussion

### New endofungal bacteria in *Rhizopus*

With the progress in exploring EFBs, an increasing number of *Burkholderia*-related endobacteria (BRE) and *Mycoplasma*-related endobacteria (MRE) have been identified in fungi, especially within the phylum Mucoromycota (Bianciotto et al., 2003; Partida-Martinez et al., 2007b; Sato et al., 2010; Okrasińska et al., 2021; Uehling et al., 2023). However, *Pandoraea sputorum*, a member of the family Burkholderiaceae, has never been previously detected within fungal hosts.

*Pandoraea sputorum* represents an emerging human pathogen known for inducing a pro-inflammatory response that can lead to lung dysfunction in individuals with cystic fibrosis (Xiao et al., 2019). This pathogenic microorganism has been exclusively isolated from respiratory tract sources (Pimentel and Macleod, 2008; Martínez-Lamas et al., 2011; Fernández-Olmos et al., 2012; Pugès et al., 2015; Kwizera et al., 2017) and blood samples (Xiao et al., 2019). Alongside this species, the pathogenic genus *Pandoraea* includes ten other species (Xiao et al., 2019). While these species have been identified in various specimens, such as sputum, blood, urine, lung tissue, and wounds, they have not been observed within fungi. Therefore, this study marks the initial proposal of the pathogenic bacterium *Pandoraea* as an EFB genus, particularly thriving within a potential pathogenic fungus of *Rhizopus arrhizus*. This underscores the heightened relevance of this genus in the field of medicine.

It has been reported that the EFB *Mycetohabitans rhizoxinica* plays a role in enhancing sporulation in the host fungus *Rhizopus microsporus* (Partida-Martinez et al., 2007c). In this study, we detected EFB *M. endofungorum* in the host fungus *R. arrhizus*, a species closely related to *R. microsporus* (Liu et al., 2008). The cured *R. arrhizus* exhibited impaired growth and an inability to produce sporangiospores, underscoring the essential role of EFB *M. endofungorum* in the growth and reproduction of *R. arrhizus*. Attempts to restore sporulation through co-culturing on LB plates were unsuccessful, likely attributed to the limited infectivity of EFB03829 on the host. Laser-mediated microinjection (Partida-Martinez et al., 2007c) emerges as a potential superior method for the reintroduction of *M. endofungorum* into its host *R. arrhizus*.

### Gene structure and specific genes of the two EFBs

Based on the complete assembly genome sequences available in the NCBI database^14^ for the *Burkholderia* genus, it is observed that their genome sizes span a range from 5.23 Mb to 10.63 Mb. Notably, the genome of *Mycetohabitans endofungorum* EFB03829 is markedly reduced, measuring only 3.64 Mb (composed of a 2,660,040 bp of chromosome, 800,149 bp of plasmid 1, and 181,953 bp of plasmid 2). This represents a significant reduction when compared to its *Burkholderia* spp. counterparts. While the genome of *Pandoraea sputorum* EFB03792 (5.85 Mb) does not exhibit notable streamlining when compared to other strains of the same species of *P. sputorum*, which range from 5.74 Mb to 6.45 Mb (see footnote 14). The prevailing consensus is that obligate endosymbionts undergo genome reduction as an adaptation to their reliance on host-derived resources (Uehling et al., 2023). In contrast, facultative endosymbiotic bacteria are generally not subject to genome reduction (Baltrus et al., 2017). Both *M. endofungorum* and *P. sputorum* are facultative endosymbiotic bacteria, demonstrating the ability to thrive not only within fungal mycelia but also on artificial media. Surprisingly, *M. endofungorum* EFB03829 exhibited a remarkable genome reduction.

According to Salvioli et al. (2017), the genome of *Mycetohabitans rhizoxinica* includes toxin-antitoxin modules (TAs), which involve in modulating growth under stress conditions and promoting survival in host cells. *M. endofungorum* also harbors TAs in its genome, potentially influencing the regulation of fungal endobacteria life (Lackner et al., 2011b; Salvioli et al., 2017). The genomes of both *M. endofungorum* and *Pandorea sputorum* strains encompass genes associated with virulence factors. And *M. endofungorum* has demonstrated the ability to produce the toxin rhizonin, exhibiting significant nonspecific hepatotoxicity (Partida-Martinez et al., 2007a). Previous research has indicated that the two *R. arrhizus* strains under investigation can undergo fermentation to generate glucose and lactic acid for food fermentation purposes (Liu et al., 2022). Consequently, the potential EFBs may pose a risk of toxin production in fermented foods.

The analyses of collinearity indicated a more pronounced change in the gene structure of *M. endofungorum* EFB03829 compared to *P. sputorum* EFB03792 (Figure 5). Furthermore, *M. endofungorum* EFB03829 possesses a significantly higher number of mobile elements (1,517) than *M. endofungorum* HKI456 (392) and *P. sputorum* (60–111; Table 1). The repeated insertion and loss of mobile elements, including prophages, can contribute to genome reduction and alterations in gene structure (Vale et al., 2022). Thus, the substantial presence of mobile genetic elements in *M. endofungorum* EFB03829 is implicated in its genome reduction and structural changes.

CAZymes, or Carbohydrate-Active Enzymes, play a key role in metabolism, involved in the synthesis, modification, and degradation of carbohydrates, including polysaccharides, glycoproteins, and glycolipids (Drula et al., 2022). Our results suggested that the newly sequenced genomes were annotated several CE10 family genes, suggesting their involvement in the metabolism of various compounds in the host *R. arrhizus*, including drugs, pesticides, and lipids.

The type III secretion system (T3SS) plays a vital role in maintaining symbiosis and is highly conserved in the genomes of Gram-negative pathogenic or symbiotic bacteria (Deng et al., 2017), such as endosymbiont *Candidatus* Glomeribacter gigasporarum associated with the arbuscular mycorrhizal fungus (AMF) *Gigaspora margaritain* (Ghignone et al., 2012) and *Burkholderia rhizoxinica* in the zygomycetous fungus *Rhizopus microsporus* (Lackner et al., 2011a). In our study, we predicted 74 and 24 genes related to T3SS in *P. sputorum* EFB03792 and *M. endofungorum* EFB03829, respectively, indicating their role as symbiotic bacteria with *Rhizopus arrhizus*. The identification of these specific genes in our study contributes to a better understanding of the mechanisms underlying the actions of symbiotic bacteria in fungi during invasion and colonization stages.

## Conclusion

This study presents a comprehensive investigation of two bacterial species, *Pandoraea sputorum* EFB03792 and *Mycetohabitans endofungorum* EFB03829, in association with *Rhizopus arrhizus* strains based on live/dead staining, FISH, TEM, and 16S rDNA sequencing. The wild-type *R. arrhizus* strains underwent more than 22 sub-cultures on a medium containing antibiotics. The results showed that *M. endofungorum* could control the sporulation of *R. arrhizus*, while *P. sputorum* had no significant effect on the morphology of *R. arrhizus*. The genome sequencing results indicate that *M. endofungorum* EFB03829 underwent genome reduction, resulting in a smaller genome size compared to *P. sputorum* EFB03792. Despite its reduced genome, EFB03829 contains more mobile genetic elements. Gene annotation revealed the presence of toxin genes in both EFBs. This raises potential safety concerns for food fermentation involving *R. arrhizus*, as the presence of toxin genes in these endofungal bacteria may pose risks during the fermentation process. The study provides valuable insights into the interactions between EFBs and *R. arrhizus*, highlighting the need for careful consideration of safety aspects in food fermentation processes involving these microorganisms.

## Data availability statement

The datasets presented in this study can be found in online repositories. The names of the repository/repositories and accession number(s) can be found at: https://www.ncbi.nlm.nih.gov/genbank/, PRJNA1046224.

## Author contributions

X-LL: Conceptualization, Data curation, Methodology, Writing – original draft, Writing – review & editing. HZ: Conceptualization, Data curation, Investigation, Methodology, Software, Writing – review & editing. Y-XW: Formal analysis, Investigation, Writing – review & editing. X-YeL: Formal analysis, Investigation, Writing – review & editing. YJ: Formal analysis, Investigation, Writing – review & editing. M-FT: Formal analysis, Investigation, Writing – original draft, Writing – review & editing. X-YoL: Funding acquisition, Methodology, Project administration, Writing – review & editing.

## Footnotes

^1^ https://www.uniprot.org

^2^ https://www.ncbi.nlm.nih.gov/protein/

^3^ http://pfam.xfam.org

^4^ https://www.kegg.jp

^5^ https://geneontology.org

^6^ http://www.cazy.org

^7^ https://www.ncbi.nlm.nih.gov/research/cog

^8^ https://card.mcmaster.ca

^9^ http://www.mgc.ac.cn/VFs

^10^ http://phaster.ca/

^11^ https://www-is.biotoul.fr/

^12^ https://www.pathogenomics.sfu.ca/islandviewer/

^13^ https://bioinfogp.cnb.csic.es/tools/venny/index.html

^14^ https://www.ncbi.nlm.nih.gov/genome

## References

[ref1] Andrews S. (2010). FastQC: a quality control tool for high throughput sequence data

[ref2] Arendt K. R. Hockett K. L. Araldi-Brondolo S. J. Baltrus D. A. Arnold A. E. (2016). Isolation of endohyphal bacteria from foliar ascomycota and in vitro establishment of their symbiotic associations. Appl. Environ. Microbiol. 82, 2943–2949. doi: 10.1128/AEM.00452-16, PMID: 26969692 PMC4959084

[ref3] Baltrus D. A. Dougherty K. Arendt K. R. Huntemann M. Clum A. Pillay M.. (2017). Absence of genome reduction in diverse, facultative endohyphal bacteria. Microb. Genom. 3:e000101. doi: 10.1099/mgen.0.000101, PMID: 28348879 PMC5361626

[ref4] Bankevich A. Nurk S. Antipov D. Gurevich A. A. Dvorkin M. Kulikov A. S.. (2012). SPAdes: a new genome assembly algorithm and its applications to single-cell sequencing. J. Comput. Biol. 19, 455–477. doi: 10.1089/cmb.2012.0021, PMID: 22506599 PMC3342519

[ref5] Becard G. Fortin J. (1988). Early events of vesicular-arbuscular mycorrhiza formation on Ri T-DNA transformed roots. New Phytol. 108, 211–218. doi: 10.1111/j.1469-8137.1988.tb03698.x, PMID: 33874168

[ref6] Bianciotto V. Lumini E. Bonfante P. Vandamme P. (2003). *Candidatus* glomeribacter gigasporarum gen. Nov., sp. nov., an endosymbiont of arbuscular mycorrhizal fungi. Int. J. Syst. Evol. Microbiol. 53, 121–124. doi: 10.1099/ijs.0.02382-0, PMID: 12656162

[ref7] Birol D. Gunyar O. A. (2021). Investigation of presence of endofungal bacteria in *Rhizopus* spp. isolated from the different food samples. Arch. Microbiol. 203, 2269–2277. doi: 10.1007/s00203-021-02251-4, PMID: 33638021

[ref8] Bolger A. M. Lohse M. Usadel B. (2014). Trimmomatic: a flexible trimmer for illumina sequence data. Bioinformatics 30, 2114–2120. doi: 10.1093/bioinformatics/btu170, PMID: 24695404 PMC4103590

[ref9] Bonfante P. Desirò A. (2017). Who lives in a fungus? The diversity, origins and functions of fungal endobacteria living in mucoromycota. ISME J. 11, 1727–1735. doi: 10.1038/ismej.2017.21, PMID: 28387771 PMC5520026

[ref10] Buchfink B. Xie C. Huson D. H. (2015). Fast and sensitive protein alignment using DIAMOND. Nat. Methods 12, 59–60. doi: 10.1038/nmeth.3176, PMID: 25402007

[ref11] Büttner H. Pidot S. J. Scherlach K. Hertweck C. (2023). Endofungal bacteria boost anthelminthic host protection with the biosurfactant symbiosin. Chem. Sci. 14, 103–112. doi: 10.1039/d2sc04167g PMC9769094 36605741

[ref12] Caporaso J. G. Lauber C. L. Walters W. A. Berg-Lyons D. Huntley J. Fierer N.. (2012). Ultra-high-throughput microbial community analysis on the Illumina hiseq and miseq platforms. ISME J. 6, 1621–1624. doi: 10.1038/ismej.2012.8, PMID: 22402401 PMC3400413

[ref13] Cappelli A. Damiani C. Capone A. Bozic J. Mensah P. Clementi E.. (2023). Tripartite interactions comprising yeast-endobacteria systems in the gut of vector mosquitoes. Front. Microbiol. 14:1157299. doi: 10.3389/fmicb.2023.1157299 37396392 PMC10311912

[ref14] Cheng Y. Gao Y. Liu X. Y. Wang G. Y. Zhang G. Q. Gao S. Q. (2017). Rhinocerebral mucormycosis caused by *Rhizopus arrhizus* var. tonkinensis. J. Mycol. Médicale 27, 586–588. doi: 10.1016/j.mycmed.2017.10.001, PMID: 29122529

[ref15] Cheng S. Jiang J. W. Tan L. T. Deng J. X. Liang P. Y. Su H.. (2022). Plant growth-promoting ability of mycorrhizal Fusarium strain kb-3 enhanced by its IAA producing endohyphal bacterium, *Klebsiella aerogenes*. Front. Microbiol. 13:855399. doi: 10.3389/fmicb.2022.855399, PMID: 35495715 PMC9051524

[ref16] Darling A. C. E. Mau B. Blattner F. R. Perna N. T. (2004). Mauve: multiple alignment of conserved genomic sequence with rearrangements. Genome Res. 14, 1394–1403. doi: 10.1101/gr.2289704, PMID: 15231754 PMC442156

[ref17] Deng W. Marshall N. C. Rowland J. L. McCoy J. M. Worrall L. J. Santos A. S.. (2017). Assembly, structure, function and regulation of type III secretion systems. Nat. Rev. Microbiol. 15, 323–337. doi: 10.1038/nrmicro.2017.20, PMID: 28392566

[ref18] Desirò A. Faccio A. Kaech A. Bidartondo M. I. Bonfante P. (2015). Endogone, one of the oldest plant-associated fungi, host unique mollicutes-related endobacteria. New Phytol. 205, 1464–1472. doi: 10.1111/nph.13136, PMID: 25345989

[ref19] Deveau A. Bonito G. Uehling J. Paoletti M. Becker M. Bindschedler S.. (2018). Bacterial–fungal interactions: ecology, mechanisms and challenges. FEMS Microbiol. Rev. 42, 335–352. doi: 10.1093/femsre/fuy008, PMID: 29471481

[ref20] Dogra S. Arora A. Aggarwal A. Passi G. Sharma A. Singh G.. (2022). Mucormycosis amid covid-19 crisis: pathogenesis, diagnosis, and novel treatment strategies to combat the spread. Front. Microbiol. 12:794176. doi: 10.3389/fmicb.2021.794176, PMID: 35058909 PMC8763841

[ref21] Drula E. Garron M. Dogan S. Lombard V. Henrissat B. Terrapon N. (2022). The carbohydrate-active enzyme database: functions and literature. Nucleic Acids Res. 50, D571–D577. doi: 10.1093/nar/gkab1045, PMID: 34850161 PMC8728194

[ref22] Estrada-De Los Santos P. Palmer M. Chavez-Ramirez B. Beukes C. Steenkamp E. T. Briscoe L.. (2018). Whole genome analyses suggests that *Burkholderia* sensu lato contains two additional novel genera (*Mycetohabitans* gen. Nov., and *Trinickia* gen. Nov.): implications for the evolution of diazotrophy and nodulation in the *Burkholderiaceae*. Genes. 9:389. doi: 10.3390/genes9080389, PMID: 30071618 PMC6116057

[ref23] Fernández-Olmos A. Morosini M. I. Lamas A. García-Castillo M. García-García L. Cantón R.. (2012). Clinical and microbiological features of a cystic fibrosis patient chronically colonized with *Pandoraea sputorum* identified by combining 16S rRNA sequencing and matrix-assisted laser desorption ionization-time of flight mass spectrometry. J. Clin. Microbiol. 50, 1096–1098. doi: 10.1128/JCM.05730-11, PMID: 22170922 PMC3295115

[ref24] Ghasemi S. Harighi B. Ashengroph M. (2023). Biosynthesis of silver nanoparticles using *Pseudomonas canadensis*, and its antivirulence effects against *Pseudomonas tolaasii*, mushroom brown blotch agent. Sci. Rep. 13:3668. doi: 10.1038/s41598-023-30863-x 36871050 PMC9985599

[ref25] Ghignone S. Salvioli A. Anca I. Lumini E. Ortu G. Petiti L.. (2012). The genome of the obligate endobacterium of an AM fungus reveals an interphylum network of nutritional interactions. ISME J. 6, 136–145. doi: 10.1038/ismej.2011.110, PMID: 21866182 PMC3246228

[ref26] Guo H. J. Glaeser S. P. Alabid I. Imani J. Haghighi H. Kampfer P.. (2017). The abundance of endofungal bacterium *Rhizobium radiobacter* (syn. *Agrobacterium tumefaciens*) increases in its fungal host *Piriformospora indica* during the tripartite sebacinalean symbiosis with higher plants. Front. Microbiol. 8:629. doi: 10.3389/fmicb.2017.00629, PMID: 28450855 PMC5390018

[ref27] Guo Y. Narisawa K. (2018). Fungus-bacterium symbionts promote plant health and performance. Microbes Environ. 33, 239–241. doi: 10.1264/jsme2.ME3303rh, PMID: 30270261 PMC6167116

[ref28] Hamza A. A. Gunyar O. A. (2022). Functional properties of *Rhizopus oryzae* strains isolated from agricultural soils as a potential probiotic for broiler feed fermentation. World J. Microbiol. Biotechnol. 38:41. doi: 10.1007/s11274-021-03225-w 35018552

[ref29] Hoffman M. T. Arnold A. E. (2010). Diverse bacteria inhabit living hyphae of phylogenetically diverse fungal endophytes. Appl. Environ. Microbiol. 76, 4063–4075. doi: 10.1128/AEM.02928-09, PMID: 20435775 PMC2893488

[ref30] Ibrahim A. S. Gebremariam T. Liu M. Chamilos G. Kontoyiannis D. Mink R.. (2008). Bacterial endosymbiosis is widely present among zygomycetes but does not contribute to the pathogenesis of mucormycosis. J. Infect. Dis. 198, 1083–1090. doi: 10.1086/591461, PMID: 18694335 PMC2729545

[ref31] Itabangi H. Sephton-Clark P. C. S. Tamayo D. P. Zhou X. Starling G. P. Mahamoud Z.. (2022). A bacterial endosymbiont of the fungus *Rhizopus microsporus* drives phagocyte evasion and opportunistic virulence. Curr. Biol. 32, 1115–1130. doi: 10.1016/j.cub.2022.01.028, PMID: 35134329 PMC8926845

[ref32] Itabangi H. Sephton-Clark P. C. S. Zhou X. Insua I. Probert M. Correia J.. (2019). A bacterial endosymbiont enables fungal immune evasion during fatal mucormycete infection. bioRxiv 101:584607. doi: 10.1101/584607

[ref33] Izumi H. Anderson I. C. Alexander I. J. Killham K. S. Moore E. (2006). Endobacteria in some ectomycorrhiza of scots pine (*Pinus sylvestris*). FEMS Microbiol. Ecol. 56, 34–43. doi: 10.1111/j.1574-6941.2005.00048.x, PMID: 16542403

[ref34] Kwizera R. Parkes-Ratanshi R. Page I. D. Sekaggya-Wiltshire C. Musaazi J. Fehr J.. (2017). Elevated aspergillus-specific antibody levels among HIV infected ugandans with pulmonary tuberculosis. BMC Pulm. Med. 17:149. doi: 10.1186/s12890-017-0500-9 29162063 PMC5699185

[ref35] Lackner G. Moebius N. Hertweck C. (2011a). Endofungal bacterium controls its host by an hrp type III secretion system. ISME J. 5, 252–261. doi: 10.1038/ismej.2010.126 20720578 PMC3105691

[ref36] Lackner G. Moebius N. Partida-Martinez L. P. Boland S. Hertweck C. (2011b). Evolution of an endofungal lifestyle: deductions from the *Burkholderia rhizoxinica* genome. BMC Genomics 12:210. doi: 10.1186/1471-2164-12-210 21539752 PMC3102044

[ref37] Lastovetsky O. A. Krasnovsky L. D. Qin X. Gaspar M. L. Gryganskyi A. P. Huntemann M.. (2020). Molecular dialogues between early divergent fungi and bacteria in an antagonism versus a mutualism. MBio 11:e02088. doi: 10.1128/mbio.02088-20 32900811 PMC7482071

[ref38] Letunic I. Bork P. (2019). Interactive tree of life (iTOL) v4: recent updates and new developments. Nucleic Acids Res. 47, W256–W259. doi: 10.1093/nar/gkz239, PMID: 30931475 PMC6602468

[ref39] Liu X. Y. Huang H. Zheng R. Y. (2008). Delimitation of Rhizopus varieties based on IGS rDNA sequences. Sydowia 60, 93–112. doi: 10.1074/jbc.271.14.8126

[ref40] Liu X. L. Ju X. Jia B. S. Qiao T. Y. Liu X. Y. (2022). The correlation of fermentation metabolites with sporulation capability and variety in *Rhizopus arrhizus*. Acta Microbiol Sin. 62, 1131–1149. doi: 10.13343/j.cnki.wsxb.20210409

[ref41] Liu Z. Sun X. Liu X. L. Jia B. S. Liu X. Y. (2019). Research progress on endofungal bacteria. Mycosystema 38, 1581–1599. doi: 10.13346/j.mycosystema.190286

[ref42] MacDonald R. Chandler M. R. (1981). Bacterium-like organelles in the vesicular-arbuscular mycorrhizal fungus glomus caledonius. New Phytol. 89, 241–246. doi: 10.1111/j.1469-8137.1981.tb07486.x

[ref43] Martínez-Lamas L. Rabade Castedo C. Martín Romero Domínguez M. Barbeito Castiñeiras G. Palacios Bartolomé A. Pérez Del Molino Bernal M. L. (2011). Colonización por *Pandoraea sputorum* en un paciente con fibrosis quística. Arch. Bronconeumol. 47, 571–574. doi: 10.1016/j.arbres.2011.06.015, PMID: 21908092

[ref44] Mondo S. J. Lastovetsky O. A. Gaspar M. L. Schwardt N. H. Barber C. C. Riley R.. (2017). Bacterial endosymbionts influence host sexuality and reveal reproductive genes of early divergent fungi. Nat. Commun. 8:1843. doi: 10.1038/s41467-017-02052-8 29184190 PMC5705715

[ref45] Mondo S. J. Toomer K. H. Morton J. B. Lekberg Y. Pawlowska T. E. (2012). Evolutionary stability in a 400-million-year-old heritable facultative mutualism. Evolution 66, 2564–2576. doi: 10.1111/j.1558-5646.2012.01611.x, PMID: 22834753

[ref46] Mosse B. (1970). Honey-coloured, sessile endogone spores: II. Changes in fine structure during spore development. Arch. Microbiol. 74, 129–145. doi: 10.1007/BF00446901

[ref47] Muszewska A. Okrasinska A. Steczkiewicz K. Drgas O. Orlowska M. Perlinska-Lenart U.. (2021). Metabolic potential, ecology and presence of associated bacteria is reflected in genomic diversity of mucoromycotina. Front. Microbiol. 12:636986. doi: 10.3389/fmicb.2021.636986, PMID: 33679672 PMC7928374

[ref48] Naumann M. Schussler A. Bonfante P. (2010). The obligate endobacteria of arbuscular mycorrhizal fungi are ancient heritable components related to the Mollicutes. ISME J. 4, 862–871. doi: 10.1038/ismej.2010.21, PMID: 20237515

[ref49] Niehs S. P. Scherlach K. Dose B. Uzum Z. Stinear T. P. Pidot S. J.. (2022). A highly conserved gene locus in endofungal bacteria codes for the biosynthesis of symbiosis-specific cyclopeptides. PNAS Nexus. 1, 1–8. doi: 10.1093/pnasnexus/pgac152 PMC9802438 36714835

[ref50] Okrasińska A. Bokus A. Duk K. Gęsiorska A. Sokołowska B. Miłobędzka A.. (2021). New endohyphal relationships between mucoromycota and burkholderiaceae representatives. Appl. Environ. Microbiol. 87, e02707–e02720. doi: 10.1128/AEM.02707-20 33483310 PMC8091615

[ref51] Partida-Martinez L. P. De Looss C. F. Ishida K. Ishida M. Roth M. Buder K.. (2007a). Rhizonin, the first mycotoxin isolated from the Zygomycota, is not a fungal metabolite but is produced by bacterial endosymbionts. Appl. Environ. Microbiol. 73, 793–797. doi: 10.1128/AEM.01784-06 17122400 PMC1800748

[ref52] Partida-Martinez L. P. Groth I. Schmitt I. Richter W. Roth M. Hertweck C. (2007b). *Burkholderia rhizoxinica* sp. nov. and *Burkholderia endofungorum* sp. nov., bacterial endosymbionts of the plant-pathogenic fungus *Rhizopus microsporus*. Int. J. Syst. Evol. Microbiol. 57, 2583–2590. doi: 10.1099/ijs.0.64660-0 17978222

[ref53] Partida-Martinez L. P. Hertweck C. (2005). Pathogenic fungus harbours endosymbiotic bacteria for toxin production. Nature 437, 884–888. doi: 10.1038/nature03997, PMID: 16208371

[ref54] Partida-Martinez L. P. Monajembashi S. Greulich K. O. Hertweck C. (2007c). Endosymbiont-dependent host reproduction maintains bacterial-fungal mutualism. Curr. Biol. 17, 773–777. doi: 10.1016/j.cub.2007.03.039 17412585

[ref55] Pawlowska T. E. Gaspar M. L. Lastovetsky O. A. Mondo S. J. Real-Ramirez I. Shakya E.. (2018). Biology of fungi and their bacterial endosymbionts. Annu. Rev. Phytopathol. 56, 289–309. doi: 10.1146/annurev-phyto-080417-045914, PMID: 30149793

[ref56] Pimentel J. D. Macleod C. (2008). Misidentification of *Pandoraea sputorum* isolated from sputum of a patient with cystic fibrosis and review of Pandoraea species infections in transplant patients. J. Clin. Microbiol. 46, 3165–3168. doi: 10.1128/JCM.00855-08, PMID: 18650348 PMC2546721

[ref57] Pugès M. Debelleix S. Fayon M. Mégraud F. Lehours P. (2015). Persistent infection because of *Pandoraea sputorum* in a young cystic fibrosis patient resistant to antimicrobial treatment. Pediatr. Infect. Dis. J. 34, 1135–1137. doi: 10.1097/INF.0000000000000843, PMID: 26176630

[ref58] Richter I. Radosa S. Cseresnyés Z. Ferling I. Büttner H. Niehs S. P.. (2022). Toxin-producing endosymbionts shield pathogenic fungus against micropredators. MBio 13, e01440–e01422. doi: 10.1128/mbio.01440-22 36005392 PMC9600703

[ref59] Richter I. Wein P. Uzum Z. Stanley C. E. Krabbe J. Molloy E. M.. (2023). Transcription activator-like effector protects bacterial endosymbionts from entrapment within fungal hyphae. Curr. Biol. 33, 2646–2656. doi: 10.1016/j.cub.2023.05.028, PMID: 37301202 PMC10337650

[ref60] Ronquist F. Teslenko M. Van Der Mark P. (2012). Mrbayes 3.2: efficient Bayesian phylogenetic inference and model choice across a large model space. Syst. Biol. 61, 539–542. doi: 10.1093/sysbio/sys029, PMID: 22357727 PMC3329765

[ref61] Rudramurthy S. M. Singh S. Kanaujia R. Chaudhary H. Muthu V. Panda N.. (2023). Clinical and mycologic characteristics of emerging mucormycosis agent *Rhizopus homothallicus*. Emerging Infect. Dis. 29, 1313–1322. doi: 10.3201/eid2907.221491, PMID: 37347535 PMC10310386

[ref62] Salvioli F. A. Lipuma J. Venice F. Dupont L. Bonfanteet P. (2017). The endobacterium of an arbuscular mycorrhizal fungus modulates the expression of its toxin-antitoxin systems during the life cycle of its host. ISME J. 2017, 2394–2398. doi: 10.1038/ismej.2017.84 PMC5607366 28548657

[ref63] Sato Y. Narisawa K. Tsuruta K. Umezu M. Nishizawa T. Tanaka K.. (2010). Detection of betaproteobacteria inside the mycelium of the fungus *Mortierella elongata*. Microbes Environ. 25, 321–324. doi: 10.1264/jsme2.ME10134, PMID: 21576890

[ref64] Scannerini S. Bonfante-Fasolo P. (1991). Bacteria and bacteria-like objects in endomycorrhizal fungi (Glomaceae). Margulis L. and Fester R., (Eds.) Symbiosis as a source of evolutionary innovation: Speciation and morphogenesis. Cham: The MIT Press, 273–287.

[ref65] Schüßler A. Mollenhauer D. Schnepf E. Kluge M. (1994). Geosiphon pyriforme, an endosymbiotic association of fungus and cyanobacteria: the spore structure resembles that of arbuscular mycorrhizal (AM) fungi. Botanica Acta 107, 36–45. doi: 10.1111/j.1438-8677.1994.tb00406.x

[ref66] Seemann T. (2014). Prokka: rapid prokaryotic genome annotation. Bioinformatics 30, 2068–2069. doi: 10.1093/bioinformatics/btu153, PMID: 24642063

[ref67] Stamatakis A. (2014). RAxML version 8: a tool for phylogenetic analysis and post-analysis of large phylogenies. Bioinformatics 30, 1312–1313. doi: 10.1093/bioinformatics/btu033, PMID: 24451623 PMC3998144

[ref68] Sun X. Chen W. Ivanov S. Maclean A. M. Wight H. Ramaraj T.. (2019). Genome and evolution of the arbuscular mycorrhizal fungus Diversispora epigaea (formerly glomus versiforme) and its bacterial endosymbionts. New Phytol. 221, 1556–1573. doi: 10.1111/nph.15472, PMID: 30368822

[ref69] Sward R. (1981). The structure of the spores of *Gigaspora margarita* the dormant spore. New Phytol. 87, 761–768. doi: 10.1111/j.1469-8137.1981.tb01712.x

[ref70] Takashima Y. Seto K. Degawa Y. Guo Y. Nishizawa T. Ohta H.. (2018). Prevalence and intra-family phylogenetic divergence of Burkholderiaceae-related endobacteria associated with species of Mortierella. Microbes Environ. 33, 417–427. doi: 10.1264/jsme2.ME18081, PMID: 30531154 PMC6307997

[ref71] Uehling J. Gryganskyi A. Hameed K. Tschaplinski T. Misztal P. Wu S.. (2017). Comparative genomics of *Mortierella elongata* and its bacterial endosymbiont Mycoavidus cysteinexigens. Environ. Microbiol. 19, 2964–2983. doi: 10.1111/1462-2920.13669, PMID: 28076891

[ref72] Uehling J. K. Salvioli A. Amses K. R. Partida-Martínez L. P. Bonito G. Bonfante P. (2023). Bacterial endosymbionts of Mucoromycota fungi: diversity and function of their interactions, Pöggeler S. James T. (Eds.) Evolution of fungi and fungal-like organisms. Cham: Springer International Publishing, 177–205.

[ref73] Vale F. F. Lehours P. Yamaoka Y. (2022). Editorial: the role of mobile genetic elements in bacterial evolution and their adaptability. Front. Microbiol. 13:849667. doi: 10.3389/fmicb.2022.849667, PMID: 35265063 PMC8899501

[ref74] Venkatesh N. Greco C. Drott M. T. Koss M. J. Ludwikoski I. Keller N. M.. (2022). Bacterial hitchhikers derive benefits from fungal housing. Curr. Biol. 32, 1523–1533. doi: 10.1016/j.cub.2022.02.017, PMID: 35235767 PMC9009100

[ref75] Xiao X. Tian H. Cheng X. Li G. Zhou J. Peng Z.. (2019). *Pandoraea sputorum* bacteremia in a patient who had undergone allogeneic liver transplantation plus immunosuppressive therapy: a case report. Infect. Drug Resist. 12, 3359–3364. doi: 10.2147/IDR.S227643 31695454 PMC6821047

[ref76] Yang S. Anikst V. Adamson P. C. (2022). Endofungal *Mycetohabitans rhizoxinica* bacteremia associated with *Rhizopus microsporus* respiratory tract infection. Emerging Infect. Dis. 28, 2091–2095. doi: 10.3201/eid2810.220507, PMID: 36148964 PMC9514336

[ref77] Yao L. D. Ju X. James T. Y. Qiu J. Z. Liu X. Y. (2018). Relationship between saccharifying capacity and isolation sources for strains of the *Rhizopus arrhizus* complex. Mycoscience 59, 409–414. doi: 10.1016/j.myc.2018.02.011

[ref78] Zhao H. Nie Y. Zong T. K. Wang K. Lv M. L. Cui Y. J.. (2023). Species diversity, updated classification and divergence times of the phylum Mucoromycota. Fungal Divers. 123, 49–157. doi: 10.1007/s13225-023-00525-4

[ref79] Zheng R. Y. Chen G. Q. Huang H. Liu X. Y. (2007). A monograph of *Rhizopus*. Sydowia 59, 273–372.

[ref80] Zhou Y. Wang H. Xu S. Liu K. Qi H. Wang M.. (2022). Bacterial-fungal interactions under agricultural settings: from physical to chemical interactions. Stress Biology. 2:22. doi: 10.1007/s44154-022-00046-1 37676347 PMC10442017

[ref81] Zimin A. V. Marçais G. Puiu D. Roberts M. Salzberg S. L. Yorke J. A. (2013). The masurca genome assembler. Bioinformatics 29, 2669–2677. doi: 10.1093/bioinformatics/btt476, PMID: 23990416 PMC3799473

