# Supplementary material for: Detecting and characterizing new endofungal bacteria in new hosts: Pandoraea sputorum and Mycetohabitans endofungorum in Rhizopus arrhizus
Source: Front Microbiol. 2024 Feb 29;15:1346252. doi: 10.3389/fmicb.2024.1346252 (PMC10939042; doi:10.3389/fmicb.2024.1346252)
Supplement: Supplementary file 1 [file Data_Sheet_1.ZIP › Supplementary_materials/Table S1.docx]

Supplementary Material

# Supplementary Table 1. Twenty-eight specific genes in fungal endosymbiont *Pandoraea sputorum* EFB03792 compared with free-living strains NCTC13161 and DSM21091

| ID | VFDB annotation | category |
| --- | --- | --- |
| assembly_01334 | VFG001870（gb\|YP_095392） （lspF） general secretion pathway protein F [&lt;i&gt;lsp&lt;/i&gt; T2SS （VF0154）] [Legionella pneumophila subsp. pneumophila str. Philadelphia 1] | Effector delivery system |
| assembly_01335 | VFG002048（gb\|YP_404601） （gspE） general secretion pathway protein E [T2SS （VF0333）] [Shigella dysenteriae Sd197] | Effector delivery system |
| assembly_01351 | VFG002437（gb\|YP_108306） （boaB） autotransporter protein [BoaB （VF0435）] [Burkholderia pseudomallei K96243] | Adherence |
| assembly_01352 | VFG000064（gb\|NP_880904） （bcrD） Type III secretion system LcrD homolog protein BcrD [TTSS （VF0035）] [Bordetella pertussis Tohama I] | Effector delivery system |
| assembly_01354 | VFG000060（gb\|NP_880900） （bopN） Bordetella Bsc type III secretion system secreted protein BopN [TTSS （VF0035）] [Bordetella pertussis Tohama I] | Effector delivery system |
| assembly_01355 | VFG000039（gb\|NP_880879） （bscC） Bordetella Bsc type III secretion system protein BscC [TTSS （VF0035）] [Bordetella pertussis Tohama I] | Effector delivery system |
| assembly_01357 | VFG000041（gb\|NP_880881） （bscU） Bordetella Bsc type III secretion system protein BscU [TTSS （VF0035）] [Bordetella pertussis Tohama I] | Effector delivery system |
| assembly_01358 | VFG000042（gb\|NP_880882） （bscT） Bordetella Bsc type III secretion system protein BscT [TTSS （VF0035）] [Bordetella pertussis Tohama I] | Effector delivery system |
| assembly_01359 | VFG000043（gb\|NP_880883） （bscS） Bordetella Bsc type III secretion system protein BscS [TTSS （VF0035）] [Bordetella pertussis Tohama I] | Effector delivery system |
| assembly_01360 | VFG038352（gb\|YP_001144302） （ascR） Type III secretion system inner membrane export apparatus protein AscR [T3SS （VF0479）] [Aeromonas salmonicida subsp. salmonicida A449] | Effector delivery system |
| assembly_01361 | VFG000045（gb\|NP_880885） （bscQ） Bordetella Bsc type III secretion system protein BscQ [TTSS （VF0035）] [Bordetella pertussis Tohama I] | Effector delivery system |
| assembly_01364 | VFG038348（gb\|YP_001144298） （ascN） Type III secretion system ATPase [T3SS （VF0479）] [Aeromonas salmonicida subsp. salmonicida A449] | Effector delivery system |
| assembly_01366 | VFG000411（gb\|NP_395195） （yscL） type III secretion system stator YscL [TTSS （VF0140）] [Yersinia pestis CO92] | Effector delivery system |
| assembly_01368 | VFG000051（gb\|NP_880891） （bscJ） Bordetella Bsc type III secretion system protein BscJ [TTSS （VF0035）] [Bordetella pertussis Tohama I] | Effector delivery system |
| assembly_01393 | VFG002424（gb\|YP_111499） （bimA） YadA family of bacterial autotransporter mediating actin tail formation [BimA （VF0427）] [Burkholderia pseudomallei K96243] | Adherence |
| assembly_01411 | VFG040745（gb\|YP_067537） （pat1） patatin-like phospholipase [Phospholipase A2 （VF0494）] [Rickettsia typhi str. Wilmington] | Exoenzyme |
| assembly_03089 | VFG000028（gb\|NP_882012） （brkB） serum resistance protein [Brk （VF0034）] [Bordetella pertussis Tohama I] | Adherence |
| assembly_03359 | VFG047672（gb\|YP_169957.1） （argP） amino acid antiporter [ArgP （VF0557）] [Francisella tularensis subsp. tularensis SCHU S4] | Nutritional/Metabolic factor |
| assembly_04414 | VFG000142（gb\|NP_253698） （waaC） 3-deoxy-D-manno-octulosonic-acid （KDO） transferase [LPS （VF0085）] [Pseudomonas aeruginosa PAO1] | Immune modulation |
| assembly_04451 | VFG002376（gb\|YP_001007268） （ddhB） CDP-glucose 4:6-dehydratase [O-antigen （VF0392）] [Yersinia enterocolitica subsp. enterocolitica 8081] | Immune modulation |
| assembly_04452 | VFG002377（gb\|YP_001007269） （ddhA） glucose-1-phosphate cytidylyltransferase [O-antigen （VF0392）] [Yersinia enterocolitica subsp. enterocolitica 8081] | Immune modulation |
| assembly_04751 | VFG002186（gb\|NP_816137） （cpsE） glycosyl transferase: group 2 family protein [Capsule （VF0361）] [Enterococcus faecalis V583] | Exotoxin |
| assembly_05001 | VFG047204（gb\|YP_170400.1） （wbtB） galactosyl transferase [LPS （VF0542）] [Francisella tularensis subsp. tularensis SCHU S4] | Immune modulation |
| assembly_05007 | VFG047111（gb\|YP_170393.1） （wbtH） asparagine synthase （glutamine-hydrolyzing） [LPS （VF0542）] [Francisella tularensis subsp. tularensis SCHU S4] | Nutritional/Metabolic factor |
| assembly_05008 | VFG001307（gb\|NP_644949） （cap8K） capsular polysaccharide synthesis enzyme Cap8K [Capsule （VF0003）] [Staphylococcus aureus subsp. aureus MW2] | Immune modulation |
| assembly_05009 | VFG000036（gb\|NP_878993） （bplC） lipopolysaccharide biosynthesis protein [LPS （VF0033）] [Bordetella pertussis Tohama I] | Immune modulation |
| assembly_05012 | VFG048797（gb\|YP_002920350.1） （ugd） UDP-glucose 6-dehydrogenase [Capsule （VF0560）] [Klebsiella pneumoniae subsp. pneumoniae NTUH-K2044] | Immune modulation |
| assembly_05177 | VFG045335（gb\|AHK25020） （llsB） SagB family dehydrogenase LlsB [LLS （VF0410）] [Listeria innocua SLCC6294] | Exotoxin |
