# Supplementary material for: Detecting and characterizing new endofungal bacteria in new hosts: Pandoraea sputorum and Mycetohabitans endofungorum in Rhizopus arrhizus
Source: Front Microbiol. 2024 Feb 29;15:1346252. doi: 10.3389/fmicb.2024.1346252 (PMC10939042; doi:10.3389/fmicb.2024.1346252)
Supplement: Supplementary file 1 [file Data_Sheet_1.ZIP › Supplementary_materials/Figure S1.pdf]

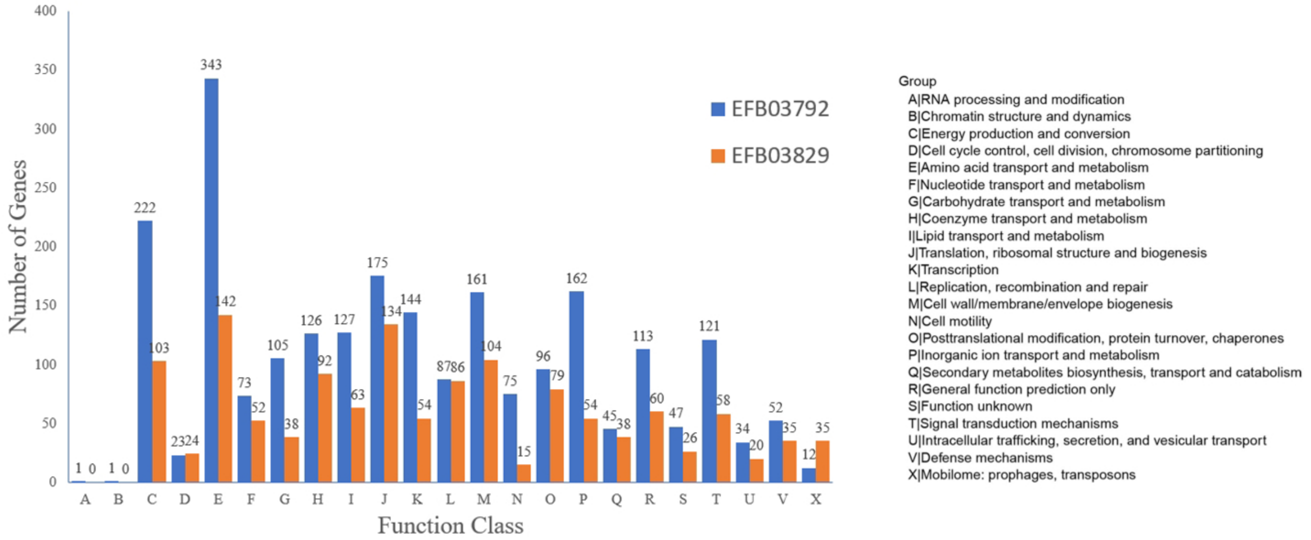

Number of Genes
Function Class
EFB03792
EFB03829
A B C D E F G H I J K L M N O P Q R S T U V X
1 0 1 0 222 103 23 24 343 142 73 52 105 38 126 92 127 63 175 134 144 54 87 86 161 104 75 15 96 79 162 54 45 38 113 60 47 26 121 58 34 20 52 35 12 35
Group
A|RNA processing and modification
B|Chromatin structure and dynamics
C|Energy production and conversion
D|Cell cycle control, cell division, chromosome partitioning
E|Amino acid transport and metabolism
F|Nucleotide transport and metabolism
G|Carbohydrate transport and metabolism
H|Coenzyme transport and metabolism
I|Lipid transport and metabolism
J|Translation, ribosomal structure and biogenesis
K|Transcription
L|Replication, recombination and repair
M|Cell wall/membrane/envelope biogenesis
N|Cell motility
O|Posttranslational modification, protein turnover, chaperones
P|Inorganic ion transport and metabolism
Q|Secondary metabolites biosynthesis, transport and catabolism
R|General function prediction only
S|Function unknown
T|Signal transduction mechanisms
U|Intracellular trafficking, secretion, and vesicular transport
V|Defense mechanisms
X|Mobilome: prophages, transposons
